# Dark biosphere: Just at the very tip of the iceberg

**DOI:** 10.1111/1462-2920.16265

**Published:** 2022-11-09

**Authors:** Cristina Escudero, Ricardo Amils

**Affiliations:** ^1^ Centro de Biología Molecular Severo Ochoa (CSIC‐UAM) Universidad Autónoma de Madrid, Cantoblanco Madrid Spain; ^2^ Centro de Astrobiología (INTA‐CSIC) Torrejón de Ardoz Spain

Nearly a century has passed since the first studies of life in the subsurface (Bastin et al., 1925; Zobel, 1938). Since then, and especially in recent decades, efforts to understand how life thrives hundreds of meters below the surface and what population dynamics operate in this environment have increased substantially.

This increased curiosity about the subsurface is not surprising. While some recent estimates of underground life indicate that at least 20% of the planet's prokaryotic biomass is subterranean, the most optimistic calculations elevate the percentage to 80% (Magnabosco et al., 2018). However, most data used in these estimates come from groundwater samples and few counts have yet been made of microorganisms populating hard rock, the main matrix on which these organisms develop (Flemming & Wuertz, 2019). More projects are needed that focus not only on the sampling and analysis of rock samples at great depths but also on the development of techniques and protocols to facilitate these tasks, since these data are crucial to more accurate estimates of subsurface Earth biomass. Subsurface environments are characterized by their heterogeneity. Geochemistry, geohydrology, the presence of organic matter or abiotic gas production greatly influences the biodiversity and metabolic activity of subsurface life (Flemming & Wuertz, 2019), so it may be inaccurate to extrapolate the scarce existing data on an environment as vast and varied as the subsurface without a proper understanding of the differences between a wide range of geological and geographical locations. The more data, the better.

There are, therefore, loads of microorganisms beneath our feet and it is important to assess the influence of their activity on the global biogeochemical cycles. After all, subterranean life does not seem to be completely independent of the surface, although it has the potential to persist in isolation without the energy input from the sun. According to recent studies, it is not yet clear whether chemoautotrophy, although widespread, is a supporting metabolism or whether subterranean communities rely on it for energy input into the systems (Meyer‐Dombard & Malas, 2022; Overholt et al., 2022; Sanz et al., 2021).

To really know the subsurface ecosystem, more holistic studies are needed, in which the production of biotic and abiotic gases, mineralogy, geohydrology, planktonic and biofilm diversity, as well as metabolic rates and activity are analysed. In fact, the most recent studies go in that direction and, thanks to them, it has been determined that metabolisms related to Fe and especially N may be key to the survival of microorganisms in the subsurface (Casar et al., 2021; Lau et al., 2014; Purkamo et al., 2020). Surprisingly, it has been estimated that, in the subsurface, a higher amount of CO_2_ can be fixed than in marine surface water (Overholt et al., 2022). This is especially relevant to the current efforts to mediate against climate change. Could the microorganisms living in underground environments hide the key to reducing greenhouse gases?

However, studies indicate that the metabolic activity rate of microorganisms in the subsurface is, in principle, significantly lower than that of the surface. Knowing the rate of growth and activity of subterranean microorganisms is, in fact, another outstanding subject for scientists working in this field. Contrary to what was thought a few years ago, even in isolated areas, where the residence time of the water is long, microorganisms are active, with their translation machinery ready and waiting for that energy input to arrive (Lopez‐Fernandez et al., 2018). Future studies combining metagenomics, proteomics, and transcriptomics together with fluorescence in situ hybridization studies using specific probes, biogeochemical analysis of the system and isotope experiments will be decisive in our understanding of the importance of the Earth's subsurface in the biogeochemical cycles at a planetary scale.

The subsurface also hides other mysteries that have fascinated the scientific community. Studies have generally focused on the microbiology of prokaryotic organisms, but both microeukaryotes and viruses, which are equally active (Lopez‐Fernandez et al., 2018), seem to play an important role in the environment. In the case of the former, the presence of potentially anaerobic or aerobic fungi, which can live in syntrophy with bacterial communities forming part of biofilms, has been confirmed. Their role in mobilizing organic matter and transforming minerals makes them a fundamental part of the mobilization of elements essential for life (Ivarsson et al., 2017). In fact, their biodiversity is curiously high, and new branches have even been very recently discovered. As for viruses, recent studies indicate that the lytic lifestyle should be deemed despite the initial thoughts considered the lysogenic life cycle as the only possibility due to the lack of energy and the supposed low metabolic activity of the host microorganisms. It has even been suggested that the heterotrophic community could be supported through the lysis of primary producers (Rahiff et al., 2021). Therefore, the use of specific techniques has been proposed to study in more depth both the fungal and viral communities, since both sampling and sample processing have been focused mainly on the detection of prokaryotes.

Another interesting unexplored avenue is the biogeography of subsurface species and how they reached it. It is very curious that microorganisms of the same species, which share more than 99% of AAI, are found in subterranean environments, hundreds of meters below the surface separated by thousands of kilometres, such as *Candidatus* Desulforudis audaxviator or *Shewanella putrefaciens* (Becraft et al., 2021; Mateos et al., 2022). Recent studies indicate that they have had a minimal evolution since Pangea, when they shared their habitat millions of years ago (Becraft et al., 2021). The subsurface therefore offers a unique opportunity to study evolution and ancient life (Drake et al., 2021).

There are still many enigmas underground, many of which pose a stimulating challenge waiting to be deciphered.

## AUTHOR CONTRIBUTIONS

**Cristina Escudero:** Conceptualization (equal). **Ricardo Amils:** Conceptualization (equal).

## CONFLICT OF INTEREST

The authors declare that they do not have any conflict of interest.
